# Supplementary material for: WordCommentsAnalyzer: A windows software tool for qualitative research
Source: F1000Res. 2018 Sep 4;7:536. Originally published 2018 May 3. [Version 2] doi: 10.12688/f1000research.14819.2 (PMC6137411; doi:10.12688/f1000research.14819.2)
Supplement: Supplementary file 3 [file f1000research-7-17614-s0002.tgz › b2a79496-4ada-47c4-9682-a99ce867836d.zip!/Eight Iranian Health Journals/Yafteh _39.docx]

© Yafteh  . This work is licensed under CC BY 4.0 (https://creativecommons.org/licenses/by/4.0/).

Volume & Issue: دوره 11 - شماره 2

Title: بررسی سطح سرمی آنتی بادی های IgM, IgG علیه کلامیدیا پنومونیه وسایرعوامل خطر در بیماران مشکوک به ایسکمی قلبی مراجعه کننده به مرکز آنژیوگرافی شهید مدنی خرم آباد

http://yafte.lums.ac.ir/article-1-849-fa.html

مقدمه: با توجه به نقش آترواسکلروزیس و بیماری های عروق کرونر در مرگ و میر انسان ها در اکثر نقاط دنیا، در این تحقیق نقش عوامل خطر مختلف در بیماران مشکوک به بیماری های قلبی مراجعه کننده به مرکز آنژیوگرافی شهید مدنی شهر خرم آباد مورد مطالعه قرار گرفته است. مواد و روش ها: در این مطالعه ابتدا بیماران مشکوک به بیماری های قلبی، با استفاده از آنژیوگرافی به دو دسته بیمار و غیر بیمار(ظاهرا سالم) تقسیم شدند. پس از پر نمودن پرسشنامه مربوط به مشخصات دموگرافیک و تغذیه ای، مقداری از خون هر دو گروه گرفته شده و تیتر آنتی بادی های IgG و IgM ضد کلامیدیا پنومونیه، و مقادیر انواع چربی، فاکتور روماتوئید، CRP و آنتی استرپتولیزین O در سرم بیماران اندازه گیری شدند. سپس اطلاعات جمع آوری شده با استفاده از تست تی و کای-دو آنالیز شدند. بحث و نتیجه گیری: درصد بالای بیماری قلبی در افراد دارای تیتر مثبت سرمی ایمونوگلبولین M ضد کلامیدیا پنومونیه می تواند نشان دهنده ارتباط بیماری فعال یا تکرار عفونت با بیماری قلبی باشد. از طرفی به نظر می آید الگوهای تغذیه ای نیز در ابتلاء به بیماری های قلبی دخیل هستند. لذا به نظر می رسد هر دو عامل میکروبی و رفتارهای غذایی افراد در گروه مورد مطالعه با بروز بیماری عروق کرونر در ارتباط باشند، که قطعا نیازمند مطالعات بیشتری است. یافته ها: کل بیماران مورد مطالعه 380 نفر بودند. حدود 120 مورد به علت مخدوش بودن اطلاعات حذف گردید. از 260 مورد 169 نفر بیمار، و 91 نفر از نظر عروق کرونر سالم تشخیص داده شدند. 143 نفر از مراجعین را مردان و 117 نفر را زنان تشکیل می دادند. 6/70% مردان(101مورد) و 58% زنان(68مورد) دچار بیماری قلبی بودند. گروه سنی 60-50 سال بیشترین(61 مورد) مبتلایان را داشت. اما گروه سنی 50-40 سال بیشترین درصد ابتلاء(8/69%) به بیماری را داشتند. مراجعین دارای گروه خونی A بیشترین درصد (1/74%) ابتلاء به بیماری را داشتند. 100 درصد کارمندان مراجعه کننده و بعد از آن 5/75% افراد مراجعه کننده با شغل دامدار دچار بیماری قلبی بودند. بین مصرف میوه، مصرف کم و زیاد گوشت و بیماری قلبی در افراد دو گروه ارتباط معنی دار بود. در این تحقیق در افراد با تیتر سرمی مثبت آنتی بادی M علیه کلامیدیا پنومونیه درصد بالایی(5/87%) از بیماری قلبی مشاهده شد.

Title: بررسی عوامل خانوادگی و شخصیتی موثر بر بزهکاری در نوجوانان بزهکار کانون اصلاح و تربیت شهر خرم آباد

http://yafte.lums.ac.ir/article-1-143-fa.html

Ø مقدمه: آسیبهای اجتماعی از معضلاتی هستند که قطعاً مانع پیشرفت و توسعه اجتماعی و هرز رفتن نیروی فعال و جمعیت جوان کشور خواهند شد و عدم توجه به این انرژی خسارات جبران ناپذیری را در پی خواهد داشت.محیط خانواده نخستین مرکزی است که کودک استعدادها و توانمندیهای خود را در آن محیط رشد و پرورش می دهد. کودکی که محیط خانواده برایش امن نباشد کمتر مقید اصول و قوانین اخلاقی است.پژوهش حاضر با هدف بررسی عوامل خانوادگی و شخصیتی موثر بر بزهکاری در نوجوانان بزهکار کانون اصلاح و تربیت شهر خرم آباد انجام گرفته است. Ø مواد و روش ها: مطالعه حاضر یک مطالعه توصیفی تحلیلی است. جامعه مورد مطالعه شامل کلیه نوجوانان مقیم در کانون اصلاح و تربیت شهر خرم آباد و دانش آموزان مشغول به تحصیل در دبیرستانهای پسرانه شهر خرم آباد می باشد. نمونه گیری در گروه مورد به روش سرشماری و در گروه شاهد به روش تصادفی خوشه ای انجام گرفت. جهت جمع آوری اطلاعات از یک پرسشنامه دو قسمتی شامل مشخصات دموگرافیک و پرسشنامه استاندارد 90 SCL استفاده گردید. Ø یافته ها: نتایج تحقیق نشان داد بین سابقه مردودی، وضعیت اقتصادی خانواده، در قید حیات بودن والدین، شغل و تحصیلات پدر، شغل و تحصیلات مادر، ارتباطات بین فردی اعضای خانواده و والدین و وجود کشمکش، درگیری و تعارض در خانواده دوگروه تفاوت معنی داری وجود دارد. همچنین نتایج تحقیق نشان داد ویژگیهای عاطفی و شخصیتی واحدهای مورد پژوهش شامل پرخاشگری، اضطراب، افسردگی، اختلال خود بیمار انگاری، اختلال وسواسی جبری، اختلال هیستریک، اختلال سایکوتیک،پارانوئیدی و فوبیادر گروه مورد و شاهد تفاوت معنی داری وجود دارد Ø بحث و نتیجه گیری: با عنایت به این امر که محیط خانواده مهمترین عامل تربیتی کودک محسوب شده و هیچ یک از آسیبهای اجتماعی خارج از تأثیر خانواده پدید نیامده است، هیچ جامعه ای نمی تواند ادعای سلامت کند مگر آن که از خانواده سالمی برخوردار باشد.

Title: شناسایی سودوموناس آئروژینوزا جدا شده از نمونه‌های تنفسی با استفاده ازواکنش زنجیره ای پلیمراز(PCR) اختصاصی ژنهای لیپوپروتئین غشای خارجی oprL و oprI و اگزوتوکسین A

http://yafte.lums.ac.ir/article-1-144-fa.html

Ø مقدمه: سودوموناس آئروژینوزا از مهمترین عوامل مرگ و میر ناشی از عفونت های بیمارستانی می باشد. با توجه به اهمیت تشخیص سریع این باکتری و اشکالات موجود در روش های بیوشیمیایی در شناسایی این باکتری، هدف بررسی توانایی آزمون PCR دو ژن اختصاصی جنس و گونه oprI و oprL و ژن اگزوتوکسین A ( toxA ) در شناسایی سودوموناس آئروژینوزا جدا شده از نمونه های تنفسی می باشد. Ø مواد و روش ها: 120 نمونه سودوموناس آئروژینوزا ازعفونتهای تنفسی جمع آوری شدند. DNA باکتری استخراج شده و آزمون PCR با استفاده از پرایمرهای اختصاصی جنس و گونه ( oprI و oprL )و پرایمرهای ژن اگزوتوکسین A ( toxA ) انجام شد. Ø یافته ها: از 120 سویه سودوموناس آئروژینوزا مورد بررسی در این مطالعه که با تستهای بیوشیمیایی جنس و گونه آنها تأیید شد 120 نمونه(100%) باروش ملکولی PCR نسبت به ژنهای اختصاصی oprI و oprL مثبت بودند. همچنین از این تعداد، 100 سویه (83%) حاوی ژن تولید کننده اگروتوکسین A بودند. Ø بحث و نتیجه گیری: جهت شناسایی سریع سودوموناس آئروژینوزا از نمونه های بالینی با توجه به نتایج بدست آمده با استفاده از PCR دو ژن oprI و oprL از حساسیت بیشتر و اختصاصیت کمتری برخوردار است در صورتی که تشخیص این باکتری با استفاده از ژن toxA دارای اختصاصیت بیشتری است.

Title: بررسی میزان آلودگی منابع آبی بیمارستانهای شهرخرم آباد به باکتری لژیونلا پنوموفیلا در سال 1387

http://yafte.lums.ac.ir/article-1-145-fa.html

مقدمه: لژیونلا باکتری آبی گرم منفی و از عوامل شایع در بروز عفونتهای بیمارستانی است. محیطهای بیمارستانی از حیث ایجاد زمینه رشد، سیستم انتقال آئروسل و افراد در معرض خطر، مکانی با پتانسیل بالا جهت رشد و شیوع این عامل است. وجود درجه حرارت مناسب در مخازن آب و سیستم توزیع آب رشد این باکتری را تشدید می کند .هدف از این پژوهش بررسی حضور لژیونلا در سیستم توزیع آب بیمارستانهای شهر خرم آباد بوده است. مواد و روش ها: نمونه برداری با تناوب 15 روزه از مخازن آب سرد و گرم بیمارستانها و همچنین شیرهای برداشت آب گرم و سرد در بخشهایی از بیمارستان که آسیب پذیری بالاتری دارند انجام شد. هر یک از نمونه ها با استفاده از فیلتراسیون غشایی تغلیظ شدند و از هر نمونه 2 پلیت بر روی محیط کشت BCYE و انتخابی GVPC کشت داده شدند و رشد باکتریها در روزهای سوم، هفتم و دهم کنترل و ثبت شد. یافته ها: در مجموع از بین 240نمونه گرفته شده از 5 بیمارستان شهر خرم آباد 7/41 درصد نمونه ها مثبت بودند. درصد نمونه های مثبت بیمارستانهای شهدای عشایر، تأمین اجتماعی، توحید، عسلیان و مدنی به ترتیب: 8/68 ، 5/45 ، 3/33 ، 1/9 و 4/36 درصد بود و میانگین کلر باقیمانده نمونه ها به ترتیب برابر 38/0،52/0، 46/0، 82/0 و 6/0 میلیگرم بر لیتر بودند. بیشترین نمونه های مثبت مربوط به سردوش آب گرم و کمترین مقدار مربوط به شیرهای آب سرد بوده اند. بحث و نتیجه گیری: با وجود اینکه همه بیمارستانهای شهر خرم آباد از آب تصفیه شده استفاده می نمایند اما از 240 نمونه جمع آوری شده 100 نمونه در بخشهای مختلف بیمارستان مثبت بودند نتایج بدست آمده ارتباط مستقیمی بین میزان کلر باقیمانده و حضور باکتری لژیونلا نشان می دهد به این ترتیب که در غلظتهای 6/0 میلیگرم در لیتر و بالاترکلر باقیمانده هیچ یک از نمونه ها مثبت نبوده اند. بنابراین معمولاً مقادیر کلر باقیمانده موجود در شبکه توزیع آب برای مقابله با لژیونلا کافی نیست.

Title: تاثیر داروی کوتریموکسازول بر فاکتورهای بیوشیمیائی خون در موش صحرائی نربالغ

http://yafte.lums.ac.ir/article-1-146-fa.html

Ø مقدمه: تری متوپریوم یک تری متوکسی بنزیل پیریمیدین محسوب می گردد که مکانیسم اثر آن مهار مسیر آنزیمی سنتز تتراهیدروفولات است. سولفامتوکسازول یک داروی آنتی باکتریال از خانواده سولفونامیدها است این دارو از سنتز دی هیدرو فولیک اسید جلوگیری می کند. تری متوپریوم که همراه با سولفونامید مصرف می گردد منجر به افزایش قابل ملاحظه ای در اثر ضد باکتریایی دارو می شود. در این تحقیق اثر مقادیر مختلف داروی کوتریموکسازول بر فاکتورهای بیوشیمیائی خون در موش صحرایی نربالغ مورد بررسی قرار گرفت. Ø مواد و روش ها: حیوانات مورد استفاده در آزمایش 50 سر موش صحرایی نر بالغ از نژاد ویستار با وزن تقریبی 10± 250گرم بود که بطور تصادفی به 5 گروه ده تایی تقسیم شدند: گروه کنترل، گروه شاهد (دریافت کننده حلال دارو یعنی پروپیلن گلیکول) و گروههای تجربی که داروی کوتریمو کسازول با مقادیر (mg/kg) 120و60و30 بصورت دهانی دریافت کردند. از تمام گروهها در پایان روز چهاردهم نمونه های خونی تهیه شد و فاکتورهای مهم بیوشیمیایی خون مانند گلوکز، کلسترول، تری گلیسیرید، اسید یوریک، کراتینین و ازت اوره خون اندازه گیری شد. نتایج حاصله به صورت مقایسه میانگین بین گروههای تجربی و کنترل با استفاده از روشهای آماری مناسب مانند ANOVA و Tukey وبا استفاده از برنامه SPSS تجزیه و تحلیل شد. Ø یافته ها: سطح سرمی کراتینین در گروههای دریافت کننده مقادیر mg/kg 120 و 60 دارو، افزایش معنی داری نسبت به گروه کنترل و شاهد نشان داد. همچنین سطح سرمی ازت اوره خون (BUN) در تمام گروههای دریافت کننده دارو نسبت به گروه کنترل و شاهد افزایش معنی دار نشان داد. سطح سرمی تری گلیسیرید خون در تمام گروههای دریافت کننده دارو نسبت به گروه کنترل و شاهد کاهش معنی دار نشان داد. کلسترول خون، تنها در گروه دریافت کننده مقدار mg/kg 120 دارو، نسبت به گروه کنترل و شاهد افزایش معنی دار نشان داد. اختلاف معنی داری در غلظت سرمی اسیدا وریک و گلوکز خون، بین گروههای تجربی، کنترل و شاهد مشاهده نشد. Ø بحث و نتیجه گیری احتمالاً داروی کوتریموکسازول از طریق کاهش جذب کلسترول کبدی و کاهش میزان هورمونهای تیروئیدی باعث افزایش کلسترول سرم می گردد. همچنین کوتریموکسازول از طریق مهار ترشح کراتینین کلیوی باعث افزایش کراتینین سرم می گردد. احتمالا این دارو از طریق ایجاد نقایص حاد کلیوی سبب افزایش BUN می گردد و با افزایش فعالیت آنزیمهای کبدی غلظت سرمی تری گلیسیرید خون را کاهش می دهد.

Title: ارتباط بین دریافت غذایی و سطح سرمی کروم با گلوکز خون، اجزای لیپیدی و HbA1c پلاسمایی در افراد مبتلا به دیابت نوع دو

http://yafte.lums.ac.ir/article-1-148-fa.html

Ø مقدمه: مطالعات نشان داده اند که ممکن است بین میزان کروم موجود در بدن و ریسک فاکتورهای بیماری دیابت ارتباطی وجود داشته باشد. هدف از مطالعه حاضر بررسی ارتباط بین دریافت غذایی و سطح سرمی کروم با اجزای لیپیدی، HbA1c و گلوکز سرمی در افراد مبتلا به دیابت نوع دو می باشد Ø مواد و روش ها: این مطالعه بر روی 30 بیمار60- 30 سال مبتلا به دیابت نوع دو (15 زن و 15 مرد) و 30 فرد سالم (15 زن و 15 مرد) صورت گرفت که از نظر سن، جنس و BMI با افراد بیمار جور شده بودند. از تمامی شرکت کنندگان سه روز یادآمد غذایی 24 ساعته گرفته شد و اخذ نمونه خون به منظور اندازه‌گیری قند خون، HbA1c ، اجزای لیپیدی و کروم سرمی به عمل آمد . Ø یافته ها: میانگین سطح سرمی کروم در گروه دیابتی ( μ gr/dl 28/0 ± 80/0) بطور معنی داری پایین تر از گروه کنترل ( μgr/dl 33 /0 ± 19/1) بود (001/0 < P ). دریافت غذایی کروم در دو گروه تفاوت معنی داری نداشت (560/0 = P ). آزمون همبستگی هیچ ارتباط معنی داری را بین سطح سرمی کروم و همچنین دریافت غذایی آن با گلوکز ناشتایی خون، HbA1c ، تری گلیسرید، کلسترول تام، HDL-C و LDL-C سرمی نشان نداد. Ø بحث و نتیجه گیری: ب علیرغم متفاوت نبودن دریافت غذایی، اختلاف معنی داری بین سطح سرمی کروم در دو گروه دیابتی و کنترل وجود داشت. کاهش سطح سرمی کروم در افراد دیابتی احتمالا بدلیل افزایش دفع ادراری این عنصر در این افراد می باشد.

Title: تاثیر غلظت های مختلف آنولیت در رشد و تقسیم سلولهای سرطانی همستر چینی

http://yafte.lums.ac.ir/article-1-149-fa.html

Ø مقدمه: در حال حاضر سرطان توسط شیمی درمانی مهار می شود ولی متاسفانه این روش دارای عوارض شدید جانبی است به همین دلیل محققین در صدند تا با کاربرد ترکیباتی، بروز این عوارض را کاهش دهند. دو نوع آب فعال شده بنام آنولیت و کاتولیت خاصیت الکتروشیمیائی و بترتیب خاصیت اکسید کنندگی و ضد میکروبی دارند. با توجه به نداشتن عوارض جانبی و خاصیت اکسیدکنندگی، هدف تحقیق بررسی تاثیرآنولیت روی رشد و تقسیم سلول های سرطانی بود. Ø مواد و روش ها : در دو آزمایش، 12 فلاکن پلاستیکی و15 فلاکن شیشه ای، سلول های سرطانی همستر چینی لاین B11dii-FAF28 کلون237 کشت داده شدند. غلظت های مختلف 7/1، 2، 5، 3/8 و 10 درصد آنولیت، وگروه کنترل سرم فیزیولوژیک، با غلظت های 2 و 5 درصد روی محیط کشت سلول ها اضافه شد. سلول های سرطانی به کمک لام هموسایتومتر و میکروسکوپ شمارش شدند. داده های بدست آمده توسط آزمون t مقایسه و با نرم افزار Excel نمودار آنها ترسیم شد.. Ø یافته ها: نتایج نشان داد که آنولیت در غلظت های مختلف تاثیر معنی داری روی رشد و تقسیم سلول ها داشته در غلظت 1.7درصد تقسیم کاهش ولی در غلظت 8.3 درصد، رشد و تقسیم سلول های سرطانی متوقف وسلول ها فیکس شدند. Ø بحث و نتیجه گیری: به نظر می رسد با توجه به مقدار کم نمک طعام موجود در آنولیت، استفاده از این ماده هیچ گونه تاثیر نامطلوبی روی سلول های سالم ندارد و می توان از بروز عوارض جانبی ممانعت نمود.

Title: بررسی نگرش دانش آموزان شهر خرم آباد نسبت به ازدواج خویشاوندی و تاثیر آن بر سلامت فرزندان

http://yafte.lums.ac.ir/article-1-150-fa.html

Ø مقدمه: توسعه و پیشرفت شرایط اقتصادی و بهداشتی کشورها از طرفی موجب کاهش مرگ و میربیماریهای عفونی و بیماریهای مرتبط با محیط و از طرف دیگر باعث افزایش سهم بیماریهای ارثی در ایجاد مرگ و میر و معلولیت در کودکان شده است. درازدواج خویشاوندی خطر تولد فرزندی با نقص مادرزادی در هر حاملگی 8-6% یا دو برابر این مقدار در کل جمعیت است. نگرش جامعه نسبت به ازدواج فامیلی می تواند تا حد زیادی در پیشگیری از اختلالات ارثی موثر باشد دانش آموزان از مهمترین گروههای موثر در این زمینه هستند، زیرا آنها والدین آینده اند و سلامت فرزندان آینده شان برای آنها مهم خواهد بود. هدف از این مطالعه تعیین نگرش دانش آموزان نسبت به ازدواج فامیلی و اثرات آن بر سلامت فرزندان بوده است. Ø مواد و روش ها : این مطالعه به صورت توصیفی – مقطعی انجام شد. نمونه شامل 1110 نفر از دانش آموزان دبیرستانی و مراکز پیش دانشگاهی شهر خرم آباد در سال 1384 بودند که به روش تصادفی خوشه ای انتخاب شدند. ابزار جمع آوری اطلاعات، پرسشنامه ای دو بخشی بود که بخش اول ویژگی های فردی و خانوادگی و بخش دوم در مورد نگرش دانش آموزان نسبت به ازدواج فامیلی و اثرات آن بر سلامت فرزندان بود. نتایج با استفاده از نرم افزار SPSS نسخه 5/11 مورد تجزیه و تحلیل قرار گرفت. Ø یافته ها: والدین دانش آموزان در 9/35% (398 نفر) ازدواج فامیلی داشتند. اکثر واحد های مورد پژوهش4/81% (904نفر) دارای نگرش بی تفاوت نسبت به ازدواج فامیلی و تاثیر آن بر سلامت فرزندان بودند. آزمون مجذور کای بین نگرش دانش آموزان نسبت به ازدواج فامیلی و اثرات منفی آن بر سلامت فرزندان با جنس(037/0= p )، رشته تحصیلی(004/0= p )، تحصیلات پدر (027/0= p )، تحصیلات مادر(024/0= p )، نسبت فامیلی والدین(002/0= p ) و ازدواج فامیلی خواهر و برادر (023/0= p ) ارتباط معنی داری نشان داد. Ø بحث و نتیجه گیری: نتایج نشان داد اکثر دانش آموزان نسبت به ازدواج خویشاوندی و تاثیر نامطلوب آن بر سلامت فرزندان و نیز نسبت به مشاوره ژنتیک و اثرات آن بر سلامت فرزندان نگرش بی تفاوت داشتند و ممکن است در آینده ازدواج خویشاوندی داشته باشند و جهت مشاوره ژنتیک هم مراجعه نکنند. بر اساس یافته های مطالعه برنامه ریزی آموزشی دقیق جهت ارتقاء دانسته ها و تغییر نگرش دانش آموزان در مورد ازدواج فامیلی و اثرات آن بر سلامت فرزندان به خصوص برای دختران دارای والدین با سطح تحصیلات پایین و دخترانی که والدینشان ازدواج خویشاوندی داشته اند پیشنهاد می شود.

Title: تأثیر مداخلات آموزشی - حمایتی بر استرس مادران دارای نوزاد نارس و طول مدت بستری

http://yafte.lums.ac.ir/article-1-151-fa.html

Ø مقدمه: تولد نوزاد نارس شوک بسیارزیادی به والدین ایجاد می کند، مادران اضطراب ،‌عصبانیت، ‌استرس و سوگواری را تجربه می کنند ،متعاقب استرس آسیب دیدگی روانی و افسردگی ایجاد می شود علیرغم آمار بالای تولد نوزاد نارس در کشورمان و استرس بالای والدین ، مطالعه ی ثبت شده ای در زمینه استرس مادران وجود ندارد. مطالعه حاضر با هدف بررسی تأثیر مداخلات آموزشی- حمایتی بر استرس مادرانی که نوزاد آنها نارس متولد شده است و طول مدت بستری انجام شده است . Ø مواد و روش ها : پژوهش حاضر مطالعه ی نیمه تجربی است که در سال 1386، در بیمارستان آموزشی امام خمینی شهر اهواز انجام شد. در گروه مداخله (30 n= ) و گروه کنترل (30 n= ) از مادران نوزادان نارس تک قلو، بدون سابقه ی تولد نوزاد نارس به روش تصادفی انتخاب و در مطالعه شرکت کردند.سطح استرس مادران در روز اول تولد با پرسشنامه ی محقق ساخته سنجیده شد، گروه مداخله برنامه حمایتی - آموزشی شامل آموزش مراقبت از نوزاد به مادر، مشارکت مادر در مراقبت از نوزاد، پیگیری سیر درمان و اطلاع رسانی به مادر و آموزش ترخیص را دریافت کردند. آموزش برنامه در 3 – 2 جلسه با کمک فیلم آموزشی نحوه ی مراقبت از نوزاد نارس (ساخته ی پژوهشگر)و کتابچه ی آموزشی انجام شد در سن یک ماهگی نوزادان،پرسشنامه ی استاندارد تنیدگی والدینی ( PSI ) در اختیار واحدهای مورد پژوهش قرار گرفت. یافته ها از طریق نرم افزار SPSS آزمون کای دو ، من ویتنی و t مستقل مقایسه شدند. Ø یافته ها: توزیع میانگین نمرات استرس واحدهای مورد پژوهش قبل از مداخله در گروه مورد و شاهد اختلاف معنی دار آماری نشان نمی دهد (66/0 p= ) پس از مداخله آزمون آماری من ویتنی بین میانگین نمرات استرس واحدهای مورد پژوهش در دو گروه مورد و شاهد اختلاف معنی دار آماری نشان می دهد (00/0 p= ). طول مدت بستری اختلاف معنی دار اماری در دو گروه مور و شاهد نشان نداد (96/0 p= ). Ø بحث و نتیجه گیری: اجرای برنامه مداخلات آموزشی- حمایتی استرس مادران نوزاد نارس را به میزان قابل توجهی کاهش داده و روی طول مدت بستری به علت کوتاه بودن مدت مطالعه اثرگذار نبود.

Title: توسعه فناوری غشاهای صاف سازی جهت حذف رنگزای راکتیو از فاضلاب صنـایع نساجی

http://yafte.lums.ac.ir/article-1-152-fa.html

Ø Ø مقدمه: رنگزای راکتیو از جمله رنگزای بکار رفته در صنایع نساجی هستند که به دلیل وزن مولکولی بالا و ساختار شیمیایی پیچیده ای که دارند، قابلیت تجزیه پذیری آن ها ناچیز بوده، و بنابراین با روش های معمول تصفیه حذف نمی گردند . هدف از این پژوهش، مطالعه روی کارایی فرایند صافسازی غشایی درحذف مواد رنگزای راکتیو در پساب حاصل از فرآیندهای نساجی می باشد. Ø مواد و روش‌ها: روش انجام تحقیق با توجه به ماهیت آن بر پایه داده‌های آزمایشگاهی، از نوع مطالعات بنیادی - کاربردی است . درمرحله اول، قابلیت تجزیه پذیری بیولوژیکی مواد رنگزای راکتیو توسط روش زاهن - والینز ( ایزو9888 مورد ارزیابی قرارگرفت . در مرحله بعد، کارایی فرایندهای صافسازی غشایی با استفاده از غشاهایی با مدول مارپیچی، دو غشاء نانوفیلتر (NF) با MWCO ، 300 و 600 دالتون به ترتیب از جنس پلی‌آمید (PPT) و سلولز استات بلند (PWB) و غشاء اسمز معکوس (Ro) با MWCO 50 دالتون از جنس پلی‌آمید مطالعه شد . برای انجام این بررسی، 4 نوع نمونه رنگ راکتیو با غلظتهای 80، 100، 250، 500 و 1000 میلی‌گرم در لیتر و همچنین نمونه‌هایی از کارخانجات مختلف نساجی، از غشاها عبور داده شد . در هر مرحله، فلاکس جریان عبوری از غشاها در دما و فشارهای مختلف در مدت زمان30 تا 60 دقیقه تعیین گردید . همچنین در هر مرحله نمونه هایی برداشت شده، و توسط دستگاه اسپکتروفتومتر UV-VIS ، به روش ADMI ، میزان رنگ در ورودی وخروجی اندازه گیری گردید . همچنین درصد حذف رنگ نیز محاسبه شد . Ø یافته‌ها: درصد حذف COD و رنگ در روش تجزیه بیولوژیکی به ترتیب 25 و5/12درصد، ومیزان ADMI ورودی وخروجی به ترتیب، 2354و2211 به دست آمد . درصد حذف COD و رنگ برای غشاهای نانوفیلتر 300 و 600 دالتون و غشاء اسمز معکوس به ترتیب برابر بود با(33%و36% : ) ، (29% و33%)، (45%،99%). (6/99% و45% ) همچنین مقادیر ADMI برای ورودی و خروجی از نانوفیلتر 300 و 600 دالتون و اسمز معکوس به ترتیب برابر (1220و1625)،(502و1625)، (103و125) بدست آمد. Ø بحث و نتیجه‌گیری: با توجه به آزمایش‌های انجام شده، فشار و دمای مناسب جهت داشتن بهترین راندمان حذف برای غشاهای نانوفیلتر 300 و 600 دالتون فشار 4 بار و دمای ˚C 35-30 می‌باشد . بر اساس اطلاعات به دست آمده، می‌توان چنین نتیجه‌گیری کرد که غلظت نمونه‌گیری تاثیر چندانی درکارآیی غشاء و افزایش فلاکس جریان ندارد . همچنین با کاربرد غشاء اسمز معکوس درصد حذف رنگ بیش از 99%بدست آمد، که نشان دهنده راندمان بالای حذف می‌باش. بنابراین فرایند صافسازی غشایی به کمک اسمز معکوس می تواند روش مناسبی برای حذف مواد رنگزا از فاضلاب صنایع نساجی پس از انجام پیش تصفیه روی فاضلاب این صنایع می باشد.
